# Selective inhibition of mitochondrial sodium-calcium exchanger protects striatal neurons from α-synuclein plus rotenone induced toxicity

**DOI:** 10.1038/s41419-018-1290-6

**Published:** 2019-01-28

**Authors:** Guendalina Bastioli, Silvia Piccirillo, Pasqualina Castaldo, Simona Magi, Alessandro Tozzi, Salvatore Amoroso, Paolo Calabresi

**Affiliations:** 1 Neurological Clinic, Department of Medicine, University of Perugia, Santa Maria della Misericordia Hospital, via Gambuli, 1, 06132 Perugia, Italy; 2 IRCCS Santa Lucia Foundation, Laboratory of Neurophysiology, via del Fosso di Fiorano, 64, 00143 Rome, Italy; 3 Department of Biomedical Sciences and Public Health, School of Medicine, University Politecnica delle Marche, via Tronto 10, 60126 Ancona, Italy; 4 Department of Experimental Medicine, Section of Physiology and Biochemistry, University of Perugia, via Gambuli, 1, 06132 Perugia, Italy

## Abstract

Progressive accumulation of α-synuclein (α-syn) and exposure to environmental toxins are risk factors that may both concur to Parkinson’s disease (PD) pathogenesis. Electrophysiological recordings of field postsynaptic potentials (fEPSPs) and Ca^2+^ measures in striatal brain slices and differentiated SH-SY5Y cells showed that co-application of α-syn and the neurotoxic pesticide rotenone (Rot) induced Ca^2+^ dysregulation and alteration of both synaptic transmission and cell function. Interestingly, the presence of the mitochondrial NCX inhibitor CGP-37157 prevented these alterations. The specific involvement of the mitochondrial NCX was confirmed by the inability of the plasma membrane inhibitor SN-6 to counteract such phenomenon. Of note, using a siRNA approach, we found that NCX1 was the isoform specifically involved. These findings suggested that NCX1, operating on the mitochondrial membrane, may have a critical role in the maintenance of ionic Ca^2+^ homeostasis in PD and that its inhibition most likely exerts a protective effect in the toxicity induced by α-syn and Rot.

## Introduction

Parkinson’s disease (PD) is a multifactorial neurodegenerative disorder mainly characterized by the damage of neurons of basal ganglia and four cardinal motor symptoms such as bradykinesia, rigidity, resting tremor, and postural instability. These pathological features are induced by the slow and progressive death of dopaminergic neurons of the substantia nigra^1^. The histopathology of PD is also characterized by the presence of Lewy bodies, which are mainly composed of aggregates of the α-synuclein (α-syn) protein^2^. Accordingly, many studies showed that in PD patients the presence of α-syn is increased in the brain^3,4^. Mutations and multiplication of the α-syn gene (SNCA) are associated with familial PD^5^. Several studies have tried to understand the role of fibrillary and oligomeric forms of α-syn on neuronal damage^6^. Conversely, only a few studies are available on the mechanisms underlying α-syn-induced synaptic dysfunction. The striatum, a subcortical nucleus receiving major excitatory inputs from the cortex and the thalamus, is a brain region particularly involved in PD. We previously demonstrated that exogenous α-syn application, applied at nanomolar concentrations, directly affects striatal neurotransmission by targeting *N*-methyl-D-aspartate (NMDA) receptors and reducing synaptic currents^7^. This α-syn-triggered NMDA receptor-dependent alteration might in turn participate in intracellular spreading of α-syn, also affecting NCX1 function expressed in the striatum at the mitochondrial level. It has also been reported that α-syn alters the hippocampal synaptic transmission in normal rodents^8^.

Accumulation of α-syn in human dopaminergic neurons leads to different neurotoxic mechanisms such as the reduction of mitochondrial complex I activity, the increase of production of reactive oxygen species, and the deregulation of mitochondrial Ca^2+^ levels^9,10^. Emerging findings suggest that exposure to environmental toxins, such as pesticides, can increase the levels of α-syn^11^. Among these drugs, rotenone (Rot), an inhibitor of mitochondrial respiratory chain complex I, induces a parkinsonian-like phenotype in rats through α-syn accumulation, caspase activation, dose-dependent ATP depletion, and oxidative damage^12–14^. In vitro studies have shown that acute application of Rot on rat striatal slices induces a widespread degeneration of striatal neurons^13^. Moreover, experiments on human neuroblastoma SH-SY5Y cells reported that combined exposition to α-syn and Rot deregulates Ca^2+^ homeostasis by opening voltage-gated Ca^2+^ channels^15,16^.

Indeed, the deregulation of Ca^2+^ homeostasis, both at cytosolic and mitochondrial levels, might contribute to the pathophysiology of neurodegenerative diseases including PD^17–20^. Both in in vitro and in vivo models of PD, it has been demonstrated that NCLX is an essential regulator of mitochondrial Ca^2+^ homeostasis^17,18^. However, several studies seem to indicate that other transporters may also play a role^21,22^. Among them, Na^+^-Ca^2+^ exchanger (NCX) has recently gained much attention. NCX is a key regulator of ionic homeostasis at both the plasma membrane and mitochondrial levels, as it can facilitate Na^+^ and Ca^2+^ flow in bidirectional way^23,24^. NCX family encompasses three members NCX1, NCX2, and NCX3 differently expressed in the brain^25,26^. NCX1 is particularly expressed in the basal ganglia where it represents a key player in controlling ionic homeostasis at both plasma membrane and mitochondrial levels in the striatum^19,25,26^. A role of plasma membrane NCX in the pathogenesis of PD has also been suggested^27^. In particular, the administration of an inhibitor of plasma membrane NCX attenuated the degeneration of dopaminergic neurons in a PD model^27^.

The aim of the present study was to analyze the involvement of both plasma membrane and mitochondrial NCX1 in the toxicity induced by combined application of α-syn and Rot in different neuronal models. Specifically, as in vitro model we used SH-SY5Y human neuroblastoma cells differentiated with retinoic acid to a neuron-like state; as ex vivo model we used striatal slices from adult rats.

## Materials and methods

### Animals

For electrophysiological experiments we used 2–3-month-old male Wistar rats (Charles River, Calco, Italy). All experimental procedures were conducted in conformity with the European Directive 2010/63/EU, in accordance with protocols approved by the Animal Care and Use Committee at the University of Perugia. All efforts were made to minimize the number of animals used as well as their suffering.

### Electrophysiology

The brain was rapidly removed and coronal corticostriatal slices (270 μm) were cut in artificial cerebrospinal fluid (aCSF) solution (in mM: 126 NaCl, 2.5 KCl, 1.2 MgCl_2_, 1.2 NaH_2_PO_4_, 2.4 CaCl_2_, 10 glucose, and 25 NaHCO_3_) using a vibratome. The slices were maintained in aCSF, bubbled with an O_2_ 95% and CO_2_ 5% gas mixture (pH = 7.4) at room temperature (RT). Single coronal slices including the cortex and the striatum were transferred to a recording chamber and submerged in a continuously flowing aCSF (33 °C; 2.5–3 ml/min) bubbled with a 95% O_2_–5% CO_2_ gas mixture^28^. Glutamatergic field excitatory postsynaptic potentials (fEPSPs) were evoked every 10 s by means of a bipolar electrode connected to a stimulation unit (Grass Telefactor) and located in the white matter between the cortex and the striatum to activate glutamatergic fibers. The recording borosilicate glass electrode filled with 2 mM NaCl (resistance 10–15 MΩ) was placed in the dorsolateral striatum. All drugs were dissolved in aCSF and bubbled with O_2_ 95% and CO_2_ 5% gas mixture during all the experiments. Then, 10 µM SN-6^29,30^ or 3 µM CGP-37157^31^ was sent in perfusion alone for 20 min and in co-application with 0.3 µM Rot. The slices treated with 3 nM α-syn were incubated for 1 h before the application of Rot.

### Cell culture

Human neuroblastoma cell line SH-SY5Y was obtained from American Type Culture Collection (ATCC CRL-2266). SH-SY5Y cells were cultured in 100 ml Petri dishes using Eagle’s minimum essential medium (MEM)/Nutrient Mixture Ham’s F-12 (1:1) media supplemented with 10% fetal bovine serum (FBS), 100 U/ml penicillin, and 100 μg/ml streptomycin. The cell culture medium was replaced every 2 days. The cells were maintained in a humidified incubator at 37 °C and 5% CO_2_.

Differentiation into neuron-like cells was achieved by treatment with 10 µM all-*trans* retinoic acid (RA)^32,33^ that was added to the cell culture medium every 3 days for 1 week prior to performing the experiments.

### Silencing of NCX1 expression

RNA interference (RNAi) was performed as described earlier^34,35^ with minor modifications. Specifically, silencing of NCX1 isoform was performed according to Qiagen manufacturer’s instruction using HiPerfect Transfection Kit (Qiagen) and FlexiTube small interference RNA (siRNA) for NCX1 (Qiagen, Hs_SLC8A1_9), and FlexiTube siRNA for NCX3 (Qiagen Hs_SLC8A3_7). The validated irrelevant Allstars siRNA (Qiagen) was used as a negative control. Target sequences of the FlexiTube NCX1 siRNA was Hs_SLC8A1_9 (5′-CAGGCCATCTTCTAAGACTGA-3′), and of the NCX3 siRNA sequences was Hs_ SLC8A3_7 (5′-ACCATTGGTCTCAAAGATTCA-3′). The transfection protocol was as follows: SH-SY5Y cells (200,000 cell/well) were differentiated with 10 µM RA in 6-well plates for 7 days. After differentiation protocol, SH-SY5Y cells were incubated 48 h with 2.3 ml of MEM7F-12 media containing 100 µl of MEM/F-12 (without FBS and antibiotics), 12 µl of HiPerfect Transfection Reagents, and 80 nM of siRNA oligonucleotide (each well). At 48 h after transfection, cells were subjected to specific treatments. The yield of RNA silencing was assessed by western blot analysis using specific antibody.

### Analysis of mitochondrial Ca^2+^

#### Experimental protocol for slices

Mitochondrial Ca^2+^ levels were monitored by single-cell computer-assisted video imaging using a LSM 510 confocal system (Carl Zeiss, Milan, Italy)^36^. The slices were loaded with 5 µM Rhod 2-AM (Molecular Probe, Eugene, OR) in aCSF solution bubbled with O_2_ 95% and CO_2_ 5% gas mixture for 1 h in the dark at RT^37^. The slices were then washed once in aCSF solution and placed into a perfusion chamber submerged in a continuously flowing aCSF solution (34 °C; 2.5–3 ml/min) bubbled with O_2_ 95% and CO_2_ 5% gas mixture, mounted onto the stage of an inverted Zeiss Axiovert 200 microscope. Mitochondrial Ca^2+^ levels were evaluated as fluorescence increase. Bath solution was changed with a peristaltic pump and images were acquired every 5 s. Excitation light was provided by argon laser at 488 nm and the emission was time-lapsed recorded at 505–530 nm. Analysis of fluorescence intensity was performed off-line after image acquisition, by averaging the fluorescence intensity values within selected areas overlying the cell somata as previously described^38,39^. There are 5 experimental groups: control, 3 nM α-syn, 0.3 µM Rot, 3 µM CGP-37157, α-syn plus Rot, CGP-37157, and α-syn plus Rot. Before the application of drugs to acquire a stable baseline, the slices were perfused with aCSF for 5 min, then were perfused for 25 min with aCSF to control, and for 25 min for other drugs dissolved in aCSF.

#### Experimental protocol for RA-differentiated SH-SY5Y

Mitochondrial Ca^2+^ levels were monitored by single-cell computer-assisted video imaging using a LSM 510 confocal system (Carl Zeiss, Milan, Italy)^36^. After being differentiated into neuron-like cells on 25 mm coverslip, SH-SY5Y were loaded with 5 µM Rhod 2-AM (Molecular Probe, Eugene, OR) in MEM/F-12 media for 1 h in the dark at 37 °C. Coverslips were then washed once in phosphate buffer solution (PBS), placed into a perfusion chamber mounted onto the stage of an inverted Zeiss Axiovert 200 microscope, and maintained in buffer solution (in mM: 140 NaCl, 5 KCl, 1 CaCl_2_, 0.5 MgCl_2_, 10 HEPES, 5.5 glucose, buffered to pH 7.4 with NaOH) and maintained at 37 °C using a heated microscope stage and climate box from PeCon GmbH. [Ca^2+^]_m_ was evaluated as fluorescence increase. Bath solution was changed with a peristaltic pump and images were acquired every 5 s. Excitation light was provided by argon laser at 488 nm and the emission was time-lapsed recorded at 505–530 nm. Analysis was obtained as previously described^39,40^. Pharmacological modulation of plasma membrane or mitochondrial NCX was performed by exposing to 1 µM SN-6 or 3 µM CGP-37157, respectively^35^. Before drug application, to acquire a stable baseline, RA-differentiated SH-SY5Y cells were perfused with buffer solution for 5 min and for 25 min with buffer solution to control, and for 25 min for other drugs dissolved in buffer solution.

### Antibodies

NCX1 protein was detected using a commercially available mouse monoclonal IgG antibody^39^ (R3F1, Swant, Bellinzona, Switzerland, dilution 1:50). To detect NCX3 protein we used a rabbit monoclonal IgG anitibody (95209, Swant, Bellinzona, Switzerland, dilution 1:200)^31,35^.

### Immucytochemistry

Differentiated SH-SY5Y cells were loaded with MitoTracker 300 nM (MitoTracker Red CMXRos M7512 Invitrogen)^31^ for 30 min at 37 °C and then fixed with PBS and 3.7% formaldehyde for 30 min at RT and then permeabilized with PBS-Triton X-100 for 5 min at RT. After permeabilization, cells were incubated with NCX1 or NCX3 primary antibody for 20 min. Immunoreactions were revealed by incubation with secondary antibody conjugated (Alexa Anti-Mouse 488, Alexa Anti-Rabbit 488 respectively).

### Drugs and chemicals

2-[[4-[(4Nitrophenyl) methoxy] phenyl] methyl]-4-thiazolidinecarboxylic acid ethyl ester (SN-6) and the mitochondrial NCX inhibitor 7-chloro-3,5-dihydro-5-phenyl-1H-4,1-benzothiazepine-2-on (CGP-37157) were obtained from Tocris. Human recombinant α-syn and Rot were purchased from Sigma.

### Data analysis

Data analysis was performed off-line using Clampfit 10 (Molecular Devices) and GraphPad Prism 6 (GraphPad Software Inc., San Diego, CA). Values given in the text and figures are mean ± S.E.M., where *n* represents the number of slices used for each electrophysiological recording. Changes in the evoked fEPSP amplitudes induced by drugs were expressed as a percentage of the baseline, the latter representing the normalized fEPSP mean amplitude acquired during a stable period (10 min) before drug administration. Two-way analysis of variance (ANOVA), followed by Bonferroni’s post hoc test, were used for statistical analysis. The significance level was established at *P* < 0.05.

Ca^2+^ levels data were expressed as mean ± S.E.M. The *n* represents the number of independent experiments and 100–200 cells were recorded in each different session. Values less than 0.05 were considered to be significant. Differences among means were assessed by one-way ANOVA followed by Dunnett’s post hoc test.

## Results

### Effect of pharmacological inhibition of NCX1 on electrophysiological recordings of striatal fEPSP amplitude

**Fig. 1:**
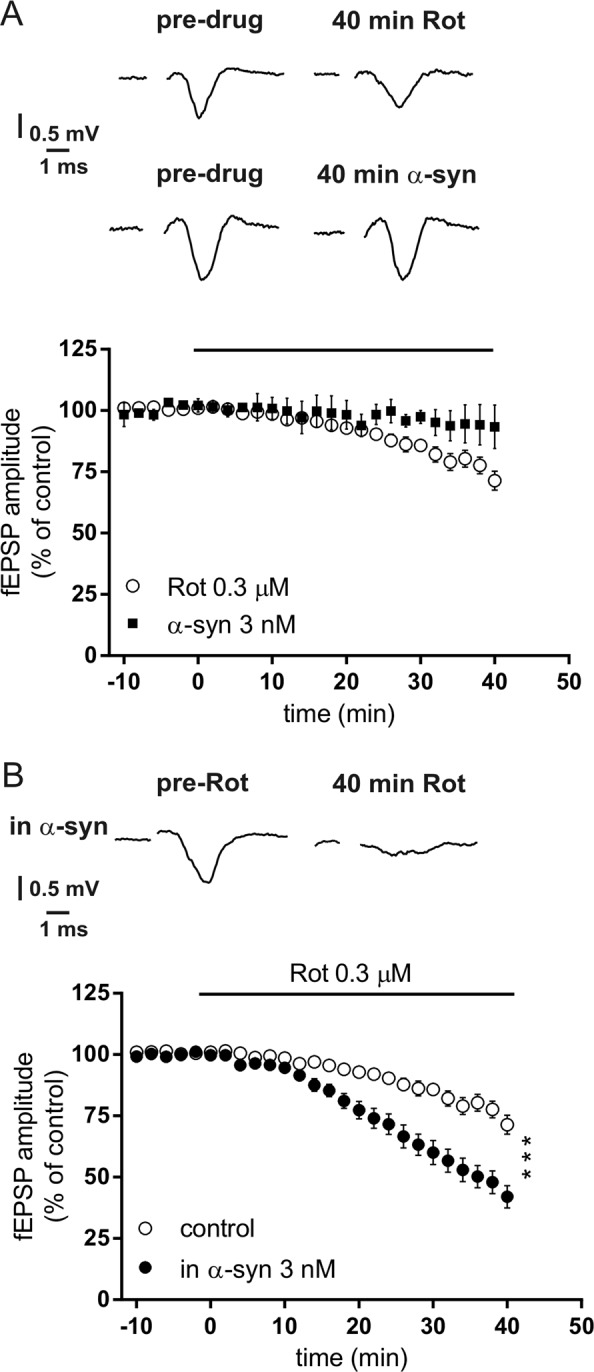
Effect of α-synuclein (α-syn) and rotenone (Rot) on striatal rat slices. **a** Time-course graph showing the field excitatory postsynaptic field potential (fEPSP) amplitude measured in striatal slices before (pre-drug) and following 3 nM α-syn (*n* = 5, filled symbols) or 0.3 µM Rot (*n* = 10, open symbols) applied for 40 min. The traces show field potentials recorded before (pre-drug) and 40 min after 3 nM α-syn or 0.3 µM Rot. **b** Time course of the fEPSP amplitude measured before and following 0.3 µM Rot applied for 40 min (*n* = 10, open symbols) and before and following 0.3 µM Rot in slices incubated for 1 h with 3 nM α-syn (*n* = 13, filled symbols) (two-way analysis of variance (ANOVA), group main factor, *F*(1, 32) = 19.79, ***P < 0.0001). The traces show field potentials recorded before and 40 min after 0.3 µM Rot in a striatal slice incubated for 1 h with 3 nM α-syn

It has been previously shown that α-syn and Rot impact neuronal cell viability, in particular they reduce mitochondrial complex I activity causing changes of Ca^2+^ levels^9,13^. In order to test whether α-syn affected per se the basal synaptic transmission, we recorded the fEPSP for 40 min in the presence of 3 nM α-syn in control striatal slices. In this condition we found no effect of α-syn on the fEPSP (Fig. 1a). Afterwards, we studied the effect of α-syn plus Rot. We recorded striatal fEPSPs in control slices and then in slices exposed for 1 h to α-syn. After acquiring a stable baseline for 10 min, 0.3 µM Rot was applied for further 40 min. We found that the fEPSP amplitude, in slices maintained in control medium (aCSF), was reduced by 30% with respect to baseline values (before Rot exposure), while the fEPSP amplitude of slices pretreated with 3 nM α-syn was reduced by 60% compared to baseline. Thus, the incubation of the slices with α-syn for 1 h significantly increased the detrimental effect of Rot on the fEPSP amplitude with respect to the effect of Rot in control slices (Fig. 1b) (*P* < 0.0001).

**Fig. 2:**
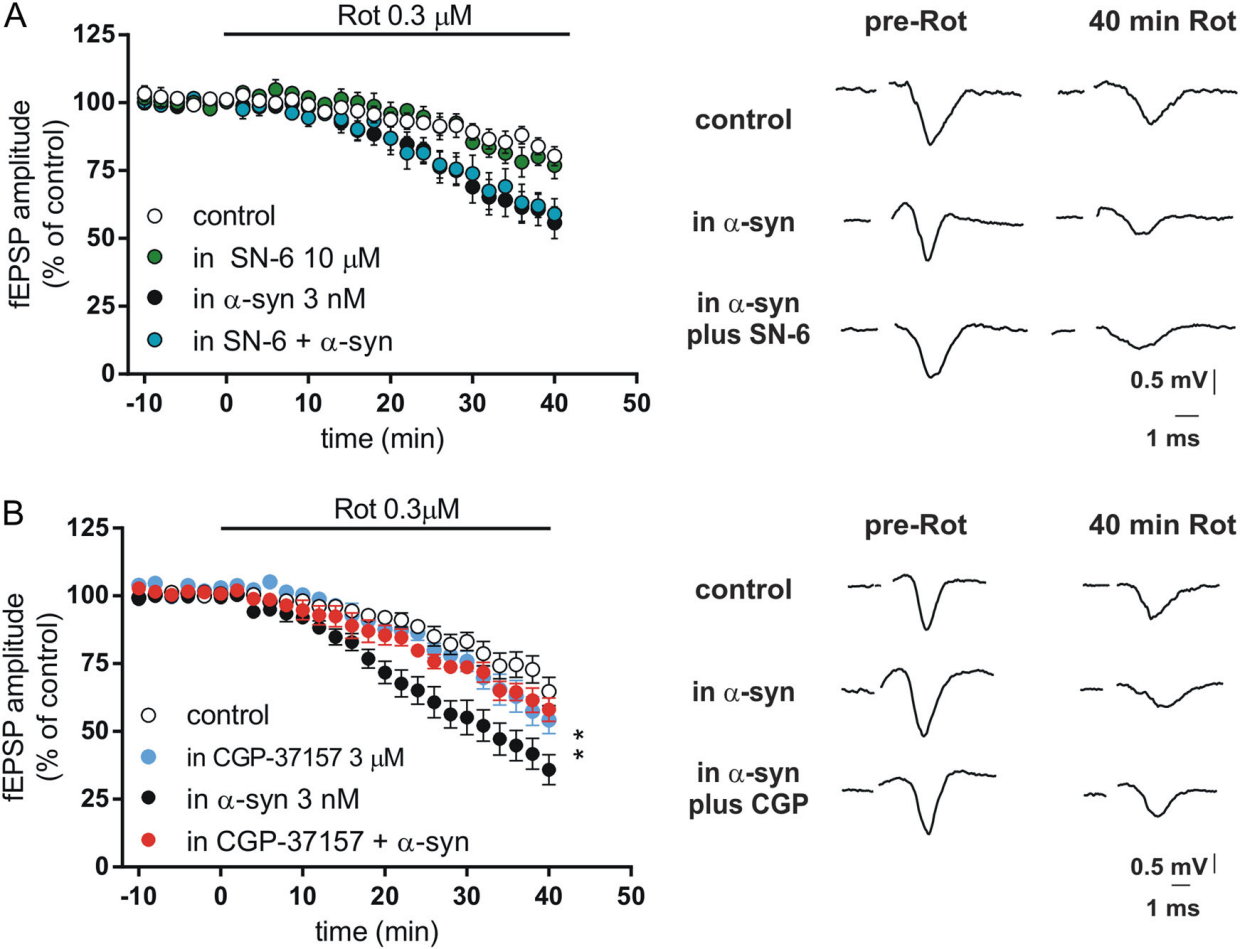
Inhibition of mitochondrial NCX1 by CGP-37157, but not membrane NCX1 by SN-6, attenuates the toxic effect of rotenone (Rot) plus α-synuclein (α-syn) in striatal slices. **a** Time course showing the field excitatory postsynaptic field potential (fEPSP) amplitude of striatal slices before and following 0.3 μM Rot application in control conditions (*n* = 10, open symbols), in the presence of 10 µM SN-6 (*n* = 4, green symbols); in slices incubated for 1 h with 3 nM α-syn (*n* = 10, filled symbols), or in presence of SN-6 in slices incubated for 1 h with α-syn (*n* = 4, cyan symbols) (SN-6+α-syn vs α-syn, two-way analysis of variance (ANOVA), group main factor *F*(1, 10) = 0.013, *P* > 0.05). Representative traces on the right show the fEPSP before (pre-Rot) and 40 min after the application of 0.3 µM Rot applied in striatal slices in different conditions. **b** Time course showing the fEPSP amplitude of slices before and following 0.3 μM Rot application in control conditions (*n* = 10, open symbols), in the presence of 3 µM CGP-37157 (*n* = 5, cyan symbols), in slices incubated for 1 h with α-syn 3 nM (*n* = 10, filled symbols), or in the presence of CGP-37157 in slices incubated for 1 h with α-syn (*n* = 6, red symbols) (CGP-37157+α-syn vs α-syn, two-way ANOVA, group main factor *F*(1, 20) = 7.853, **P < 0.01). Representative traces showing the fEPSP before (pre-Rot) and 40 min after the application of 0.3 µM Rot in different conditions

Since NCX plays a critical role in Ca^2+^ homeostasis^31^, we hypothesized that NCX1 was involved in the toxic effects exerted by α-syn plus Rot. To explore this issue, we used the plasma membrane NCX1 inhibitor SN-6 (10 µM) and the mitochondrial NCX (NCX_m_) inhibitor CGP-37157 (3 µM). SN-6 affected neither the striatal response to Rot alone nor response to the combined application of Rot and α-syn (Fig. 2a) (*P* > 0.05), suggesting that the plasma membrane NCX1 was not involved in the toxic effects of α-syn and Rot. To investigate the role of NCX_m_ isoform, striatal slices were exposed to CGP-37157 for 20 min. While this drug did not affect the toxic effect induced by Rot alone, it was able to reduce the detrimental effect produced by the combined application of α-syn and Rot on the fEPSP amplitude (Fig. 2b), suggesting a critical role for NCX_m_ in the damage induced by α-syn and Rot in our experimental model.

### Analysis of mitochondrial Ca^2+^ levels in striatal slices

**Fig. 3:**
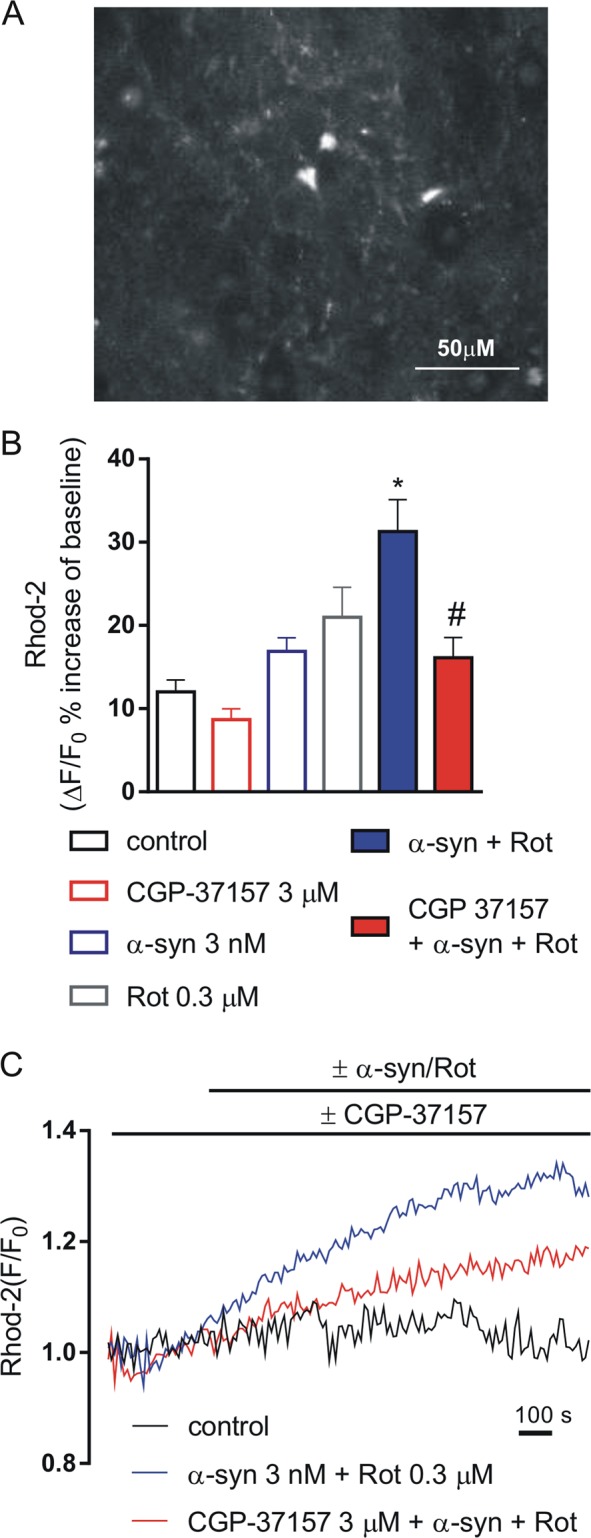
Effect of CGP-37157 on mitochondrial Ca^2+^ levels in striatal slices exposed to α-synuclein (α-syn) and rotenone (Rot). **a** Representative image of a striatal slice showing neurons loaded with Rhod 2-AM. **b** Histogram showing mitochondrial Ca^2+^ levels expressed as percentage of the fluorescence change increase (Δ*F*/*F*%) under resting conditions (open bars) and during 3 nM α-syn + 0.3 µM Rot applied alone or in combination with 3 µM CGP-37157 (filled bars). For each experimental group, Δ% values used for the statistical analysis derived from 4 independent experiments and 50–100 cells were recorded in each different session (*P < 0.001 α-syn+Rot vs all groups, ^#^*P* < 0.001 CGP-37157+α-syn+Rot vs α-syn+Rot). **c** Representative recordings of mitochondrial Ca^2+^ responses in control conditions (black line), in the presence of α-syn+Rot (blue line) and in the presence of α-syn+Rot co-applied with 3 µM CGP-37157 (red line). Fluorescence intensity values were normalized to resting fluorescence (*F*/*F*0)

It has been reported that α-syn and Rot increase mitochondrial Ca^2+^ levels and membrane depolarization of neurons^9,10,41^. Thus, we analyzed the effect of α-syn and Rot alone on mitochondrial Ca^2+^ levels of striatal neurons obtained from striatal slices preparations (Fig. 3a). We found that neither α-syn nor Rot induced per se significant changes of mitochondrial Ca^2+^ levels, while combined application of these two agents significantly increased mitochondrial Ca^2+^ levels of 31% (Fig. 3b) (*P* < 0.0001). This increase was statistically significant with respect to control and to all other groups. Interestingly, incubation with CGP-37157 was able to reduce the increase of mitochondrial Ca^2+^ levels induced by combined application of α-syn and Rot (Fig. 3b, c) (*P* < 0.001).

### Analysis of mitochondrial Ca^2+^ levels in RA-differentiated SH-SY5Y cells

The involvement of NCX on the increase of mitochondrial Ca^2+^ levels caused by the combined application of α-syn and Rot was evaluated in Rhod-2-loaded SH-SY5Y cells. We blocked plasma membrane and mitochondrial NCX1 using 1 µM SN-6 and 3 µM CGP-37157, respectively. In order to assess the role of NCX1 we used the RNAi approach to specifically silence its expression.

**Fig. 4:**
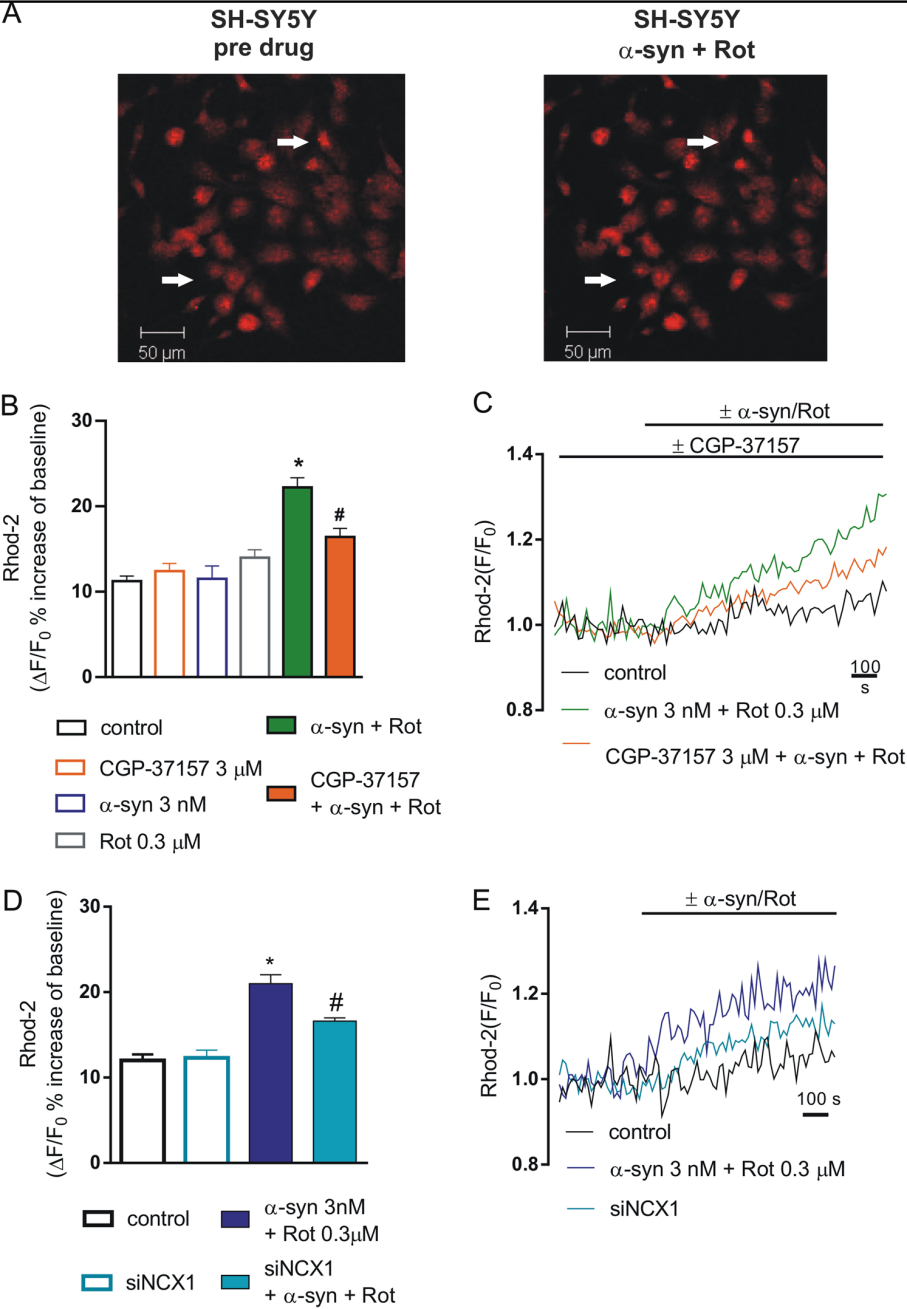
Effect of CGP-37157 and siNCX1 on mitochondrial Ca^2+^ levels in SH-SY5Y differentiated cells exposed to α-synuclein (α-syn) and rotenone (Rot). **a** Representative images of SH-SY5Y differentiated cells loaded with Rhod 2-AM before (left) and after Rot+α-syn application (right). **b** Histogram showing mitochondrial Ca^2+^ levels expressed as Δ% fluorescence increases under resting conditions (open bars) and during 3 nM α-syn + 0.3 µM Rot applied alone or in combination with 3 µM CGP-37157 (filled bars). For each experimental group (control, CGP-37157, α-syn, α-syn+Rot, CGP-37157+α-syn+Rot), Δ% values used for the statistical analysis derived from 4 independent experiments and 100–200 cells were recorded in each different session (*P < 0.0001, α-syn+Rot vs all groups; ^#^*P* < 0.001, CGP-37157+α-syn+Rot vs α-syn+Rot; CGP-37157+α-syn+Rot vs control; CGP-37157+α-syn+Rot vs α-syn). **c** Representative recordings of mitochondrial Ca^2+^ responses in control conditions (black line), in the presence of α-syn+Rot (green line) co-applied with 3 µM of CGP-37157 (orange line). **d** Histogram showing mitochondrial Ca^2+^ levels expressed as Δ% fluorescence increases in SH-SY5Y control cells, in SH-SY5Y cells treated with siNCX, in cells treated with α-syn+Rot, and in cells silenced with siNCX and α-syn+Rot. For each experimental group Δ% values used for the statistical analysis derived from 6 independent experiments and 100–200 cells were recorded in each different session (*P < 0.0001, α-syn+Rot vs. all groups; ^#^*P* < 0.001 siNCX α-syn+Rot vs all groups). **e** Representative recordings of mitochondrial Ca^2+^ responses in control conditions (black line), in the presence of α-syn+Rot (blue line), and α-syn+Rot in cells silenced with siNCX1 (cyan line)

**Fig. 5:**
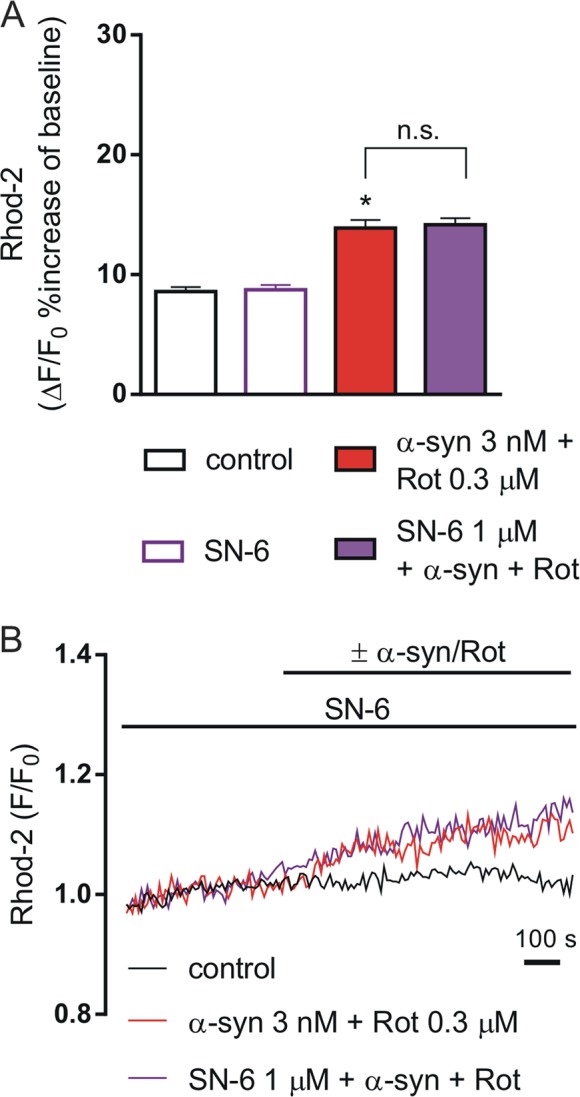
Effect of SN-6 on mitochondrial Ca^2+^ levels in SH-SY5Y differentiated cells exposed to α-synuclein (α-syn) and rotenone (Rot). **a** Histogram showing mitochondrial Ca^2+^ levels expressed as Δ% fluorescence increases under resting conditions (open bars) and during 3 nM α-syn + 0.3 µM Rot applied alone or in combination with 1 µM SN-6 (filled bars). For each experimental group, Δ% values used for the statistical analysis derived from 4 independent experiments and 50–100 cells were recorded in each different session (*P < 0.0001 α-syn+Rot vs control and control+SN-6; the α-syn+Rot group was not significantly different from α-syn+Rot+SN-6 group). **b** Representative recordings of mitochondrial Ca^2+^ responses in control conditions (black line), in the presence of α-syn plus Rot (green line) co-applied with 3 µM of SN-6 (violet line)

We found that mitochondrial Ca^2+^ levels significantly increased (22%) in the presence of α-syn plus Rot and this effect was statistically significant with respect to control and all other groups (Fig. 4a, c) (*P* < 0.0001). In line with the results obtained on fEPSP in striatal slices, SN-6 did not counteract the mitochondrial Ca^2+^ level deregulation induced by α-syn plus Rot, suggesting that plasma membrane NCX1 was not involved in such response (Fig. 5). By contrast, pretreatment with CGP-37157 reduced the effect of α-syn plus Rot on mitochondrial Ca^2+^ levels (Fig. 4b, c) (*P* < 0.001).

We also used the RNAi-mediated approach to specifically silence NCX1 expression^35^. Considering that differentiated SH-SY5Y cells express both NCX1 and NCX3 isoforms but not NCX2^35^, the specificity of NCX1 silencing towards NCX3 expression was further verified (Fig. S1). This approach specifically targeted NCX1 isoform inducing a reduction of its mitochondrial protein level without exerting any effect on NCX3 at both plasma membrane and mitochondrial levels (Fig. S1). Notably, the reduced NCX1 expression was able to prevent the increase in mitochondrial Ca^2+^ levels induced by α-syn plus Rot application (Fig. 4d, e). Overall, these findings supported the hypothesis that mitochondrial NCX1 plays an important role in the dysregulation of Ca^2+^ homeostasis induced by α-syn plus Rot.

## Discussion

The regulation of Ca^2+^ homeostasis is an important process for neuronal survival, and its dysregulation has long been recognized to play a major role in neurodegenerative diseases^38^. In the present study, using different neuronal models (namely striatal slices and RA-differentiated SH-SY5Y cells), we showed that mitochondrial NCX1 is involved in the cell toxicity induced by the concomitant exposure to α-syn and Rot. In particular, this finding is supported by three main results. Firstly, the alteration of striatal electrical activity induced by α-syn and Rot is prevented by pharmacological inhibition of mitochondrial NCX with CGP-37157, but not by using the plasma membrane NCX inhibitor SN-6. Secondly, α-syn and Rot increased mitochondrial Ca^2+^ levels in striatal slices through a mechanism that involved NCX_m_, since CGP-37157 partially prevented this response. Finally, the silencing of NCX1 in RA-differentiated SH-SY5Y cells prevented the increase of mitochondrial Ca^2+^ levels and mimicked the effect of CGP-37157.

While several studies have investigated the toxic effects induced by α-syn and Rot on neurons, including synaptic dysfunction, oxidative stress, and mitochondrial complex I deficiency^42–44^, little is known on the mechanisms underlying the convergent detrimental effects induced by the concomitant application of α-syn and Rot. Previous reports have demonstrated that in PC12 cells and in neurons of the substantia nigra, Rot raised intracellular Ca^2+^, leading to increased α-syn aggregation^45^.

Here, we presented electrophysiological evidence that in striatal rat slices, α-syn plus Rot led to enhanced neurotoxic effects causing progressive irreversible loss of excitatory striatal transmission and electrical signals. This detrimental effect could be prevented by blocking mitochondrial NCX1, suggesting that NCX_m_ could represent an important target for the control of acute synaptic dysfunction induced by the concomitant action of α-syn and Rot. Growing evidence suggest that, in the presence of toxic stimulus, the mitochondrial Ca^2+^ uptake may induce apoptosis in a variety of pathological conditions^38^. In particular, Ca^2+^ dysregulation has been previously reported in α-synopathy models of PD. Accordingly, evidence from many studies suggest that α-syn and Ca^2+^ may be related in several ways. For instance, it has been reported that α-syn induces a reduction of ΔΨ_m_ and a disruption of electronic transport complex (ETC), thus accelerating the mitochondrial permeability transition pore (mPTP) opening^10^. In addition, an increase in cytosolic Ca^2+^ levels may also occur since α-syn may form plasma membrane pores, allowing extracellular Ca^2+^ to pass into the cytosol^46^. This in turn may worsen mitochondrial function through the increased Ca^2+^ uptake^10,46^. Moreover, Angelova et al.^47^ showed that the overexpression of intracellular α-syn in neuroblastoma cells altered basal and depolarizing-stimulus-evoked Ca^2+^ signals and confirmed that α-syn per se can induce a Ca^2+^ flux across neuronal membranes through pore formation^47^. Several Ca^2+^ transporters are implicated in the ionic regulation, and between them NCXs (namely NCX1, NCX2, and NCX3^26,27^) represent critical proteins contributing to Ca^2+^ homeostasis in the brain^19,26^. Accordingly, the expression of all three NCX isoforms has been already reported in isolated mitochondria^20^. Recently, the main role of plasma membrane and mitochondrial NCX1 in controlling energy metabolism in several cell types, including neurons and astrocytes, has been demonstrated^31,48^. Interestingly, a metabolic stimulus, such as glutamate, could induce the reverse mode of NCXm, as a consequence of glutamate-induced Na^+^ influx through excitatory amino acid transporters^31^. α-Syn and Rot interact with Na^+^/K^+^-ATPase with a reduction of its ability to pump out Na^+^, thus leading to Na^+^ accumulation. Consequently, Ca^2+^ overload may occur^49,50^. In line with these observations, it is possible to speculate that under our conditions, the Na^+^ influx and the disruption of ETC may contribute to ΔΨ_m_ perturbation, ultimately forcing the mitochondrial NCX1 to work on the reverse mode and leading to Ca^2+^ increase. Our data showed that in SH-SY5Y cells, Ca^2+^ increase can be prevented by silencing of NCX1, mimicking the effect of CGP-37157 in striatal slices. An interesting study has already proposed that plasmalemmal NCX isoforms, in particular NCX2 and NCX3, contribute to mitochondrial Na^+^/Ca^2+^ exchanger in human DAergic neurons acting downstream PINK1, a pathway implicated in a recessive form of PD, preventing neuronal death induced by mitochondrial Ca^2+^ accumulation^20^. Notably, the role of another mitochondrial Ca^2+^ antiporter, NCLX, has been reported^51^. Palty et al.^17^ and Luongo et al.^18^ demonstrated that in SH-SY5Y undifferentiated cells and transgenic mice, NCLX is an essential regulator for mitochondrial Ca^2+^ homeostasis. Nevertheless, the same authors showed that the pharmacological modulation of NCLX (using CGP-37157) did not fully inhibit mitochondrial Ca^2+^ efflux. Thus, NCLX may play an important role but its contribution is not sufficient to regulate mitochondrial Ca^2+^ levels. Therefore, as suggested by several evidences, it is reasonable to hypothesize that other transporters, including NCX1, play a critical role in controlling mitochondrial Ca^2+^ homeostasis^19,21^.

In line with our findings and existing literature, we can assert the relevance of mitochondria in the pathology of PD. The involvement of mitochondrial defects in PD pathology is also supported by pharmacological evidence showing deleterious effects of inhibitors of the mitochondrial electron transport chain, such as Rot, mPTP, or paraquat^52^. Furthermore, our data confirmed that α-syn has an important role in controlling neuronal mitochondrial dynamics, and in particular in regulating Ca^2+^ signals in animal models of PD as well as in PD patients^52^.

In conclusion, our study shows for the first time a critical interaction among α-syn Rot-induced toxicity and mitochondrial NCX1, suggesting that NCX1 might represent a possible target for disease modifying therapy in PD.

## Supplementary information

Supplementary information

Fig. S1
