# Supplementary material for: Selective inhibition of mitochondrial sodium-calcium exchanger protects striatal neurons from α-synuclein plus rotenone induced toxicity
Source: Cell Death Dis. 2019 Jan 28;10(2):80. doi: 10.1038/s41419-018-1290-6 (PMC6349907; doi:10.1038/s41419-018-1290-6)
Supplement: Supplementary file 1 — Supplementary information [file 41419_2018_1290_MOESM1_ESM.docx]

**Supplementary information**

**Figure Legends**

**Fig. S1.** Analysis of the specificity of siRNA silencing for NCX1 (A) and NCX3 (B) isoforms by using immunocytochemistry technique as describes in “Materials and Methods” section. C) siRNA NCX1 specifically silenced NCX1 and did not interfere with NCX3 expression. Images are representative of three independent experiments. Scale bar 50 µM. siNCX1= siRNA for NCX1; siNCX3= siRNA for NCX3.
