# Supplementary figures and images for: Selective inhibition of mitochondrial sodium-calcium exchanger protects striatal neurons from α-synuclein plus rotenone induced toxicity
Source: Cell Death Dis. 2019 Jan 28;10(2):80. doi: 10.1038/s41419-018-1290-6 (PMC6349907; doi:10.1038/s41419-018-1290-6)

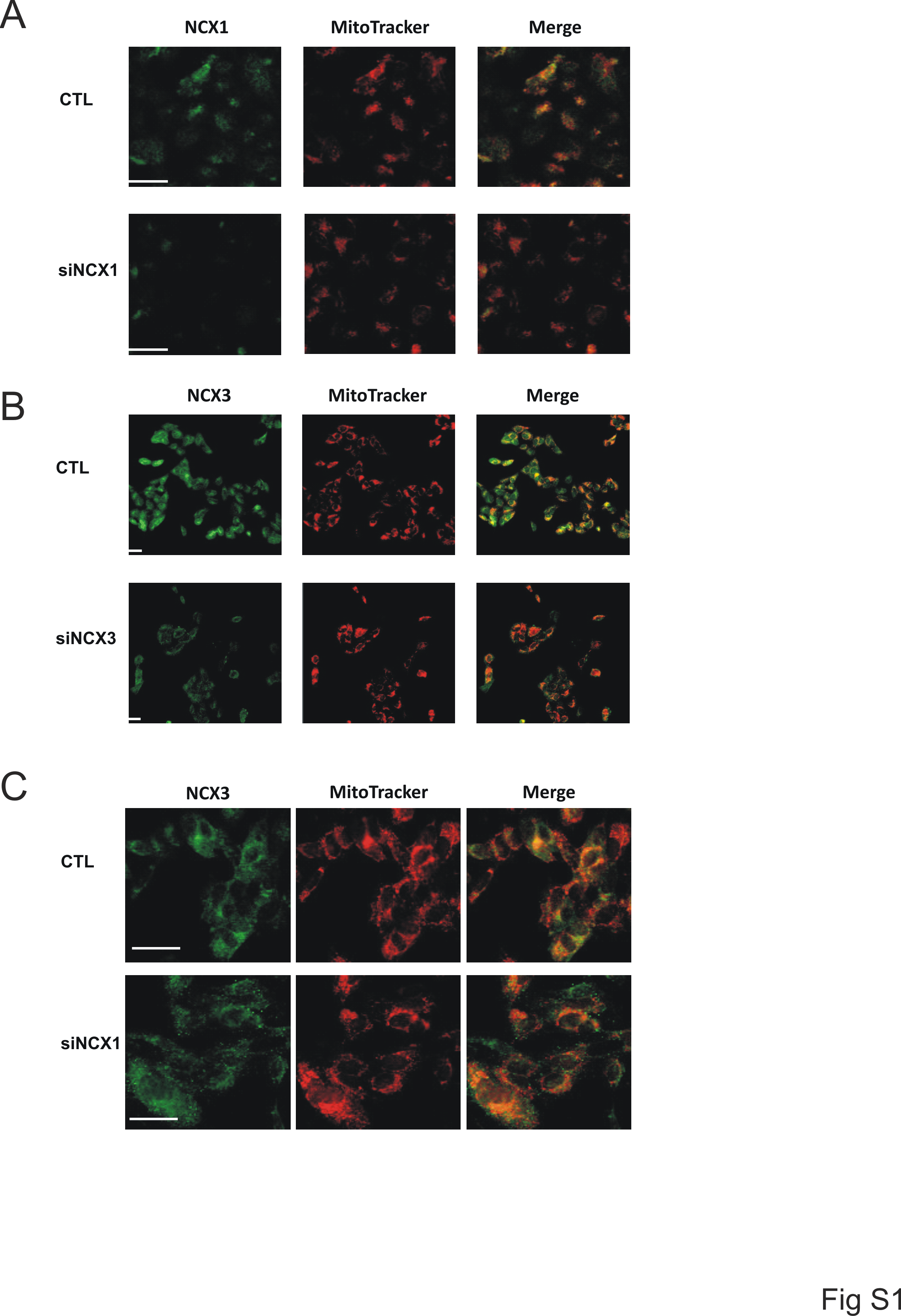

Supplement: Supplementary file 2 — Fig. S1 [file 41419_2018_1290_MOESM2_ESM.tif]
